# The Influence of Near Vision Tasks on Intraocular Pressure in Normal Subjects and Glaucoma Patients

**DOI:** 10.18502/jovr.v17i4.12350

**Published:** 2022-11-29

**Authors:** Mohammad Pakravan, Azadeh Samaeili, Hamed Esfandiari, Kiana Hassanpour, Sadid Hooshmandi, Shahin Yazdani, Farideh Sharifipour, Azadeh Doozandeh, Bahram Einollahi, Parastou Pakravan, Mohammad Hasan Shahriari, Bahareh Kheiri

**Affiliations:** ^1^Glaucoma and Neuro-Ophthalmologist, Jones Eye Institute, University of Arkansas for Medical Sciences, AR, USA; ^2^Ophthalmic Research Center, Research Institute for Ophthalmology and Vision Science, Shahid Beheshti University of Medical Sciences, Tehran, Iran; ^3^Department of Ophthalmology, Olmsted Medical Center, Rochester, MN, USA; ^4^Miller School of Miami, University of Miami, Miami, Florida; ^5^Department of Health Information Technology and Management, School of Science, Shahid Beheshti University of Medical Sciences, Tehran, Iran

**Keywords:** Accommodation, Accommodative Tasks, Intraocular Pressure, Primary Open-angle Glaucoma

## Abstract

**Purpose:**

To investigate the effect of static accommodative tasks on intraocular pressure (IOP) of glaucomatous and normal eyes.

**Methods:**

Four groups of subjects categorized as primary open-angle glaucoma (POAG), primary angle-closure suspects (PACS), normal age-matched controls, and normal young adults (NYA; age < 40 years) were enrolled. The baseline IOPs were measured after the subjects were looking at a distant target for 15 min. Static accommodation was obtained by execution of near vision tasks (reading at 33 cm in daylight [300 lux] for 60 min). IOPs were measured at 15, 30, 45, and 60 min intervals while accommodating and then measured again after 15 min of relaxing accommodation while looking at a distant target.

**Results:**

One-hundred and eighteen eyes of 98 subjects were recruited. The study groups consisted of the following categories: 25 POAG (46 eyes), 24 PACS (47 eyes), 25 matched controls (50 eyes), and 24 NYA (48 eyes). Within all groups, the mean IOP decreased throughout the accommodation period at all time points. Maximum IOP reduction after accommodation was detected at the 30-min time among the POAG subjects, at the 45-min time in the PACS and matched control groups, and at 15 min after the relaxation of accommodation in the NYA group. IOP reduction levels showed no statistically significant difference among POAG, PACS, and the normal matched groups in their response to accommodation. However, NYA had significantly lower IOP and greater IOP reduction after the resting period (relaxation of accommodation).

**Conclusion:**

Static accommodative tasks can significantly reduce IOP in normal, POAG, and PACS individuals. Encouraging glaucoma patients to practice periodical near vision tasks could be viewed as an adjunctive measure for glaucoma management.

##  INTRODUCTION

Accommodation refers to the ability of the eye to change focus from a distant object to a near object. During accommodation, ciliary muscle contraction leads to zonular fiber relaxation which is then followed by a change in lens shape where it becomes more spherical. Meanwhile, longitudinal fibers in the ciliary muscles that adhere to the scleral spur (SS) cause posterior movement of the SS during accommodation. As a result, the trabecular meshwork (TM) and adjacent Schlemm's canal expands and trabecular outflow of aqueous humor increases.^[1]^

Investigating the role of accommodation on intraocular pressure (IOP) has presented contradictory results. Static accommodation refers to focusing on a near target without changing to distance target for almost 3 min or more. While the patients look at near target and distance frequently for 3 s in repeated accommodation.^[2]^ In some reports, both static and repeated accommodations have been shown to decrease IOP in healthy individuals.^[2,3]^ The plausible mechanism could be the adjustment of elastic tissue structures of the chamber angle following numerous accommodation and ciliary muscle contractions. This modification could enhance trabecular aqueous humor drainage in a sinusoidal pattern.^[4]^ However, further studies showed that repeated accommodation does not induce a significant reduction in IOP as compared to static accommodation.^[2]^ Similarly, Baser et al^[5]^ reported that reading does not affect IOP in healthy individuals. Indeed, some studies have reported an increase in IOPs during accommodation. Ha et al^[6]^ investigated the effects of working with smartphones on the IOP in normal patients and reported an increasing effect, especially in low-light conditions. Similarly, Vera et al^[7]^ reported IOP to increase while reading both in supine and sitting positions which was greater in the sitting position.

Considering the contradictory results on the effect of engaging in near vision work on IOP and also the scarcity of studies about accommodation effects on the IOP of glaucomatous eyes, we conducted the present study. This study aims to investigate the magnitude of IOP changes induced by continuous or static accommodation during near vision work in glaucomatous and normal eyes.

##  METHODS

A total of 191 eyes of 98 patients were enrolled in the study. The Institutional Review Board of Shahid Beheshti University of Medical Science approved the protocol of the study. Our research adhered to the tenets of the Declaration of Helsinki. Informed written consent was obtained from each subject. The study groups consisted of 25 POAG subjects (46 eyes; group 1), 24 PACS subjects (47 eyes; group 2), 25 matched controls (50 eyes; group 3), and 24 normal young individuals (48 eyes; group 4). The first three groups were selected using the independent simple random sample selection technique. The fourth group were normal volunteers, young (not presbyopic and aged < 40 years) residents of the center. It was assumed that residents experienced approximately equal amounts of weekly physical activities. Diagnosis of POAG was made by the detection of glaucomatous optic neuropathy in the presence of open angles on gonioscopy and also by the lack of any evidence of secondary causes of glaucoma after baseline examination. PACS was defined when the posterior TM was not visible at 180 or more degrees of the angle during gonioscopic examination.^[8]^

Exclusion criteria were the existence of peripheral anterior synechiae (PAS), history of glaucoma surgery, ocular trauma, use of any topical or systemic medications that could affect accommodation including pilocarpine, ocular pathologies that would compromise IOP reading, high myopia (spherical equivalent refractive error >- 6.00 D), and high hyperopia (defined as spherical equivalent hyperopia of more than 4.0 D).

At baseline, the ophthalmologic examination included best-corrected visual acuity (BCVA) measurement, slit-lamp examination (Haag-Streit, Bern, Switzerland), and IOP measurement with rebound tonometry performed by a glaucoma specialist (iCare Finland Oy, Helsinki, Finland). In addition, gonioscopy with a Zeiss-style four-mirror lens (model OPDSG, Ocular Instruments, Inc., Bellevue, WA), fundus examination, perimetry (Humphrey visual field analyzer; model 750; Carl Zeiss Meditec, Dublin, California, USA), and central corneal pachymetry (Quantel medical pocket, Japan) were performed at baseline.

Using the Shaffer gonioscopy classification, a trained ophthalmologist performed the gonioscopy at × 16 magnification in a darkroom setting. The same ophthalmologist measured the IOP twice between 9 and 11 am. The ophthalmologist was masked about the patients' group. An average of two readings were recorded as the patient's IOP. If two IOPs differed by more than 2 mmHg, the measurement was repeated, and the mean IOP was recorded. To maintain a static accommodative state, patients were asked to continue their fixation on the object used for the near vision task examination with the opposite eye during IOP measurements.

After a complete explanation of the study protocol to the patients, they were seated in an upright position while the neck was in a neutral position. Patients were then required to look at a distance target (6/12 Snellen letters) for 15 min while wearing their corrective lenses. Baseline IOP was then measured. Static accommodation was achieved by close monitoring of the volunteers while they focused on a near target (reading the same text from a 19 Samsung monitor with brightness of 250 cd/m2 at 33 cm at daylight [300 lux]) for 1 hr while wearing their presbyopic glasses. iCare IOP measurements were then taken every 15 min during the 1 hr of the static accommodative task, and 15 min after focusing on a distant object in relaxed accommodative state. Before the start of the study, all subjects' IOPs were controlled either by topical medications or Yttrium Aluminum Garnet (YAG) laser peripheral iridotomy (PI) in the glaucoma cases, resulting in all IOPs becoming ≤23 mmHg.

### Statistical Analysis

To present the data, we used mean and standard deviation analysis. To compare the results among the groups, and throughout the study whenever needed, we used GEE (Generalized Estimating Equations) to consider the possible correlation of the IOP results in the eyes. The primary outcome measure was the change in IOP 15 min after the start of accommodation. The IOP values at other time intervals were considered as secondary outcome measures. To evaluate the changes within groups during the follow-up times we used paired *t*-test. In all the analyses, multiple comparison corrections were done using the Bonferroni method. All statistical analyses were performed using the SPSS software (IBM Corp. Released 2017. IBM SPSS Statistics for Windows, Version 25.0. Armonk, NY: IBM Corp.). A *P*-value < 0.05 was considered statistically significant.

##  RESULTS

One hundred and ninety-one eyes of 98 consecutive participants were enrolled in the study. The mean age of participants in groups 1 to 4 were 55.44 ± 13.96, 56.32 ± 13.79, 51.64 ± 11.1, and 29.75 ± 3.65 years, respectively.

There were no statistically significant differences among study groups 1, 2, and 3 in terms of age, refractive status, and central corneal thickness but there were statistically significant differences in group 4 [Table 1].

None of the individuals were using either pilocarpine or other systemic agents that could constrict or dilate the pupil. Eighty percent (37 eyes) of the POAG patients were on glaucoma medications that presumably do not affect accommodation.

Baseline demographic and clinical characteristics of all groups are presented in Table 1.

For all groups, the IOP was measured at baseline, and at every 15 min throughout the hour of the accommodation period. IOP was measured again, 15 min after the release of accommodation where the patients focused on a distant target.

The mean IOP decreased in all of the volunteers during the accommodating period. The mean baseline IOP ± SD was 14.43 ± 3.96, 14.11 ± 3.99, 13.62 ± 4.57, and 14.27 ± 3.98 mmHg in groups 1 to 4, respectively (*P* = 0.807).

Table 2 shows the IOP changes and differences from baseline within and among the groups. In group 1, IOP significantly decreased from baseline with a mean change of 0.98 ± 2.2 mmHg (*P* = 0.004), –1.17 ± 2.24 mmHg (*P* = 0.001), and –0.8 ± 2.46 mmHg (*P* = 0.032) at 15, 30, and 45-min time points after static accommodation.

In group 2, IOP decreased significantly from baseline at all time points throughout the accommodation. The amount of IOP decrease at each pre-determined 15 min time interval was –1.19 ± 2.09 (*P* < 0.001), –1.02 ± 2.93 (*P* = 0.021), –1.43 ± 2.89 (*P* = 0.002), and –1.17 ± 2.64 (*P* = 0.004), respectively. The IOP reduction for group 2 was also significant after 15 min of relaxation of accommodation (–1.04 ± 2.41 mmHg; *P* = 0.005). In group 3, IOP reduced throughout all examinations, however, it was significant only at the 30 and 45-min time points with a mean reduction of –1.06 ± 2.4 and –1.1 ± 2.4 mmHg, respectively (*P* = 0.003). In group 4, IOP reduction was significant at all time points within and after accommodation (–1.29 ± 2.24, –1.54 ± 2.35, –2.08 ± 2.55, –1.75 ± 2.27, and –2.15 ± 2.67, respectively, *P* < 0.001; Table 2).

**Figure 1:**
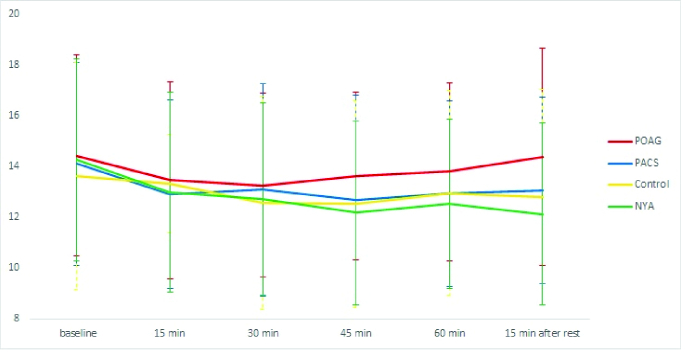
Changes in IOP between and within study groups throughout the study. Maximum IOP reduction was observed 30 min after a near task in POAG, 45 min after accommodation in PACS and control groups, and 15 min after the end of accommodation in NYA. POAG, primary open-angle glaucoma; PACS, primary angle-closure suspect; NYA, normal young adults.

Maximum IOP reduction was observed 30 min after the near vision task in group 1 (–1.17 ± 2.24 mmHg), 45 min after accommodation in groups 2 and 3 (–1.43 ± 2.89 and –1.1 ± 2.46 mmHg, respectively), and 15 min after the end of the release of accommodation in group 4 (–2.15 ± 2.67 mmHg). The mean IOPs and IOP reductions showed no statistically significant difference among the study groups in time intervals of 15, 30, 45, and 60 min after the start of the accommodation [Table 2, Figure 1].

However, normal young adults showed significantly lower mean IOP (12.13 ± 3.59, *P* = 0.04) and greater IOP reduction (–2.15 ± 2.67, *P* < 0.001) after the release of the accommodation.Utilizing multivariate analysis, only the factor of age was associated with greater IOP reduction (*P* = 0.02).

**Table 1:** The baseline demographic and clinical characteristics of the study participants.


		<@orange**Study Groups**				
	**Total**	**POAG**	**PACS**	**Control**	**NYA**	***P*** **-value**
**Sex**	M	48 (49.0%)	13 (52.0%)	12 (50.0%)	12 (48.0%)	11 (45.8%)	0.992*
	F	50 (51.0%)	12 (48.0%)	12 (50.0%)	13 (52.0%)	13 (54.2%)	
**Age Ref**	48.47 ± 15.62	55.44 ± 13.96	56.32 ± 13.79	51.64 ± 11.1	29.75 ± 3.65	< 0.001*
	0.34 ± 1.25	0.53 ± 1.36	0.66 ± 1.15	0.72 ± 1.12	-0.45 ± 0.99	< 0.001*
**CCT**	536.56 ± 73.84	549.55 ± 36.35	547.49 ± 23.97	538.71 ± 44.62	522.8 ± 13.01	< 0.001*
**Baseline IOP**	14.1 ± 4.12	14.43 ± 3.96	14.11 ± 3.99	13.62 ± 4.57	14.27 ± 3.98	0.807**
	
	
*Based on Fisher's exact test; **Based on Generalized estimating equations; Ref, refraction; CCT, central corneal thickness; POAG, primary open-angle glaucoma; PACS, primary angle-closure glaucoma; NYA, normal young adults; IOP, intraocular pressure							

**Table 2:** Changes in IOP between and within study groups throughout the study.


	**Study groups**				***P*** -value**	**Overall effect size (Cohen's d)**
	**POAG**	**PACS**	**Control**	**NYA**	
**Baseline IOP**	14.43 ± 3.96	14.11 ± 3.99	13.62 ± 4.57	14.27 ± 3.98	0.807	
**IOP at 15min**	13.46 ± 3.87	12.91 ± 3.73	13.32 ± 4.47	12.98 ± 3.93	0.884	
**IOP change at 15 min**	–0.98 ± 2.2	–1.19 ± 2.09	–0.3 ± 1.93	–1.29 ± 2.24	0.06	0.11
***P*** -within*	0.004	< 0.001	0.277	< 0.001	
**IOP at 30 min**	13.26 ± 3.62	13.09 ± 4.19	12.56 ± 4.2	12.73 ± 3.78	0.804	
**IOP change from baseline at 30 min**	–1.17 ± 2.24	–1.02 ± 2.93	–1.06 ± 2.4	–1.54 ± 2.35	0.709	0.14
***P*** -within*	0.001	0.021	0.003	< 0.001	
**IOP at 45 min**	13.63 ± 3.3	12.68 ± 4.12	12.52 ± 4.07	12.19 ± 3.62	0.198	
**IOP Change from baseline at 45 min**	–0.8 ± 2.46	–1.43 ± 2.89	–1.1 ± 2.46	–2.08 ± 2.55	0.075	0.27
***P*** -within*	0.032	0.002	0.003	< 0.001	
**IOP at 60 min**	13.8 ± 3.51	12.94 ± 3.65	12.96 ± 4.03	12.52 ± 3.33	0.323	
**IOP change from baseline at 60 min**	–0.63 ± 2.4	–1.17 ± 2.64	–0.66 ± 2.76	–1.75 ± 2.27	0.068	0.24
***P*** -within*	0.081	0.004	0.097	< 0.001	
**IOP after 15 min rest**	14.37 ± 4.28	13.06 ± 3.67	12.8 ± 4.25	12.13 ± 3.59	0.049	
**IOP Change from baseline after 15 min rest**	–0.07 ± 2.56	–1.04 ± 2.41	–0.82 ± 2.4	–2.15 ± 2.67	0.001	0.41
***P*** -within*	0.864	0.005	0.019	< 0.001	
	
	
*P-within based on Paired *t*-test; **Based on generalized estimating equation (multiple comparison correction has been done with Bonferroni method); NYA, normal young adults; POAG, primary open-angle glaucoma; PACS, primary angle-closure suspect; IOP, intraocular pressure						

##  DISCUSSION

This study found a consistent reduction in the mean IOP during static accommodation in all groups.

Our results were in line with previous studies which showed that IOP decreased during accommodation.^[9,10]^

IOP is predominantly controlled by aqueous outflow through the TM, which is augmented by traction on the SS. Increased traction on the SS enlarges the pores of the TM. There is histological, pharmacological, and electrophysiological evidence explaining the association between accommodation and IOP; there is a connection between elastin fibers in the tendons of the longitudinal fibers of ciliary muscles and elastin fibers of the TM lamellae. Ciliary muscle tendon density is highest in the juxtacanalicular tissue near the TM adjacent to the Schlemm canal (SC).^[11]^ Therefore, the presence of pilocarpine or any factors that may trigger accommodation results in ciliary muscle contractions followed by a stretch of the TM and an increase in the area of the SC, and subsequent increase in aqueous outflow and IOP reduction.^[12,13]^ However, human studies have not yet demonstrated the changes in SC and TM morphology on physiological accommodation. The mean IOP reduction during accommodating occurs between the ranges of 1.8–4.5 mmHg as shown in the previous reports.^[2,14,15,16]^ The period of IOP measurement was between 8 and 10 min immediately after accommodation in these studies which can justify a slightly higher IOP reduction as compared to our results.^[2,14,15,16]^

We found that age was significantly associated with IOP reduction in all groups. The magnitude of IOP reduction was greater in older participants during accommodation. This observation may be explained by the pore sizes in TM. It is possible that changes in normal pore sizes have a limited effect on IOP corresponding to a small age-related increase in IOP seen between 20 and 60 years of age.^[17]^ However, even a 0.25-micron change in the size of the small pore increasing with age can result in a 10 mmHg change in the IOP.^[18]^

We observed that the accommodation-induced reduction in the IOP was seen in open-angle as well as angle-closure eyes. We argue that while accommodation pushes the iridolenticular diaphragm forward and shallows the angle, its beneficial effects on TM outweigh these changes and result in IOP reduction. However, the lower IOP reduction in POAG individuals at the 60 min time point could be attributed to the stiffness of TM in this group of patients.^[19]^

In contrast to our findings, some studies demonstrated that IOP was elevated during accommodation while reading^[7]^ or while using a smartphone^[6]^ or in myopic eyes.^[20,21]^ However, this result is not consistently repeated in other studies.^[22]^ The divergent reaction of IOP to accommodation in myopic eyes could be the result of the differences in structure and function of the eyes between emmetropic and myopic subjects.^[22]^ Ahnul et al studied normal-tension glaucoma cases that were controlled by medication or filtering surgery. They found an elevation of IOP after 25 min of working on a smartphone in low-light conditions.^[23]^ However, their study varied in the type of glaucoma, the low-light conditions, the duration of the near vision task, and the use of smartphones versus computer screens as was done in the current study. Another concern that may influence outcomes is that bending the head when reading on a smartphone may be comparable to prone positioning^[24,25]^ which is different from the upright position normally applied during computer work.

Although the IOP reduction persisted throughout the accommodation and at least 15 min after accommodation in our study, it was hard to predict how long the hypotensive effect of accommodation would last especially when compared with alternative studies where IOP was mainly measured during and immediately after accommodation.^[20,21][22]^ Of note, the effect sizes of IOP estimated by Cohen's d demonstrated small to moderate differences among groups [Table 2].

One limitation of the current study is that we did not include a control group with no accommodation. Sitting upright could potentially have some hypotension effects, however, the literature review for this effect is inconclusive.^[26,27,28]^

We also did not evaluate the biometric changes of the anterior chamber to determine the interaction between IOP and changes in lens thickness and positioning, depth of the anterior chamber as well as angle parameters. In an anterior segment study, Yan et al demonstrated that the anterior chamber became shallow, the lens thickened, and the angle narrowed during accommodation.^[20]^

Another limitation of the study is that only including PACS' eyes makes it hard to estimate the reaction of eyes with complete angle-closure to accommodation. The effect of ciliary muscle contraction in the presence of PAS could be opposite to that of the open-angle or PACS; it is shown that accommodation inhibits uveoscleral outflow resulting in paradoxical IOP elevation in synechial angle closure.^[4]^ Further studies with a longer period of resting after accommodation along with analyses among different ethnicities are warranted to elucidate the duration of the effect of accommodation on IOP as well as correlations as it pertains to other ethnicities.

In summary, in this present study, IOP was shown to be significantly reduced after static accommodation for 1 hr in normal, POAG, and PACS eyes. The hypotensive effect lasted at least 15 min after the relaxation of accommodation. Encouraging glaucoma patients to perform periodic near vision tasks such as studying would not only improve knowledge on the topic but also be helpful as an adjunctive measure in glaucoma management.

##  Financial Support and Sponsorship 

None.

##  Conflicts of Interest

Authors have no proprietary or commercial interest in any materials discussed in this article.

## References

[B1] Lütjen-Drecoll E (1999). Functional morphology of the trabecular meshwork in primate eyes. Prog Retin Eye Res.

[B2] Jenssen F, Krohn J

[B3] Priluck AZ, Hoie AB, High RR, Gulati V, Ghate DA (2020). Effect of near work on intraocular pressure in emmetropes. J Ophthalmol.

[B4] Johnstone MA (2004). The aqueous outflow system as a mechanical pump: Evidence from examination of tissue and aqueous movement in human and non-human primates. J Glaucoma.

[B5] Baser G, Karahan E, Bilgin S, Unsal U

[B6] Ha A, Kim YK, Park YJ, Jeoung JW, Park KH (2018). Intraocular pressure change during reading or writing on smartphone. PloS One.

[B7] Vera J, Redondo B, Molina R, Cárdenas D, Jiménez R (2020). Acute intraocular pressure responses to reading: The influence of body position. J Glaucoma.

[B8] Weinreb  RN, Friedman  DS

[B9] Mauger RR, Likens CP, Applebaum M (1984). Effects of accommodation and repeated applanation tonometry on intraocular pressure. Am J Optom Physiol Opt.

[B10] Read SA, Collins MJ, Becker H, Cutting J, Ross D, Savill AK, et al (2010). Changes in intraocular pressure and ocular pulse amplitude with accommodation. Br J Ophthalmol.

[B11] Park CY, Lee JK, Kahook MY, Schultz JS, Zhang C, Chuck RS (2016). Revisiting ciliary muscle tendons and their connections with the trabecular meshwork by two photon excitation microscopic imaging. Invest Ophthalmol Vis Sci.

[B12] Barany E (1966). The mode of action of miotics on outflow resistance. A study of pilocarpine in the vervet monkey Cercopithecus ethiops Trans Ophthalmol Soc UK.

[B13] Chowdhury UR, Hann CR, Stamer WD, Fautsch MP (2015). Aqueous humor outflow: Dynamics and disease. Invest Ophthalmol Vis Sci.

[B14] Armaly MF, Burian HM (1958). Changes in the tonogram during accommodation. AMA Arch Ophthalmol.

[B15] Armaly MF, Jepson NC (1962). Accommodation and the dynamics of the steady-state intraocular pressure. Invest Ophthalmol Vis Sci.

[B16] Cassidy L, Delaney Y, Fitzpatrick P, Blake J (1998). Effect of accommodation on intraocular pressure in glaucomatous eyes. Irish J Med Sci.

[B17] Abu-Hassan DW, Acott TS, Kelley MJ (2014). The trabecular meshwork: A basic review of form and function. J Ocul Biol.

[B18] Schachar RA (2006). The mechanism of accommodation and presbyopia. Int Ophthalmol Clin.

[B19] Wang K, Li G, Read AT, Navarro I, Mitra AK, Stamer WD, et al (2018). The relationship between outflow resistance and trabecular meshwork stiffness in mice. Sci Rep.

[B20] Yan L, Huibin L, Xuemin L (2014). Accommodation-induced intraocular pressure changes in progressing myopes and emmetropes. Eye.

[B21] Young FA

[B22] Liu Y, Lv H, Jiang X, Hu X, Zhang M, Li X (2015). Intraocular pressure changes during accommodation in progressing myopes, stable myopes and emmetropes. PloS One.

[B23] Ha A, Kim YK, Kim J-S, Jeoung JW, Park KH

[B24] Cheng MA, Todorov A, Tempelhoff R, McHugh T, Crowder CM, Lauryssen C (2001). The effect of prone positioning on intraocular pressure in anesthetized patients. Anesthesiology.

[B25] Ozcan MS, Praetel C, Bhatti MT, Gravenstein N, Mahla ME, Seubert CN (2004). The effect of body inclination during prone positioning on intraocular pressure in awake volunteers: A comparison of two operating tables. Anesth Analg.

[B26] Najmanová E, Pluháček F, Haklová M (2020). Intraocular pressure response affected by changing of sitting and supine positions. Acta Ophthalmol.

[B27] Mayalı H, Tekin B, Kayıkçıoğlu ÖR, Kurt E, İlker SS (2019). Evaluation of the effect of body position on intraocular pressure measured with rebound tonometer. Turk J Ophthalmol.

[B28] Uzlu D, Akyol N, Türk A, Gürsoy N, Somuncu AM, Oruç Y (2020). Effect of body position on intraocular pressure measured by rebound tonometer in healthy children. Turk J Ophthalmol.

